# Evaluation of Horizontal Stresses in Soil during Direct Simple Shear by High-Resolution Distributed Fiber Optic Sensing

**DOI:** 10.3390/s19173684

**Published:** 2019-08-24

**Authors:** Assaf Klar, Michael Roed, Irene Rocchi, Ieva Paegle

**Affiliations:** 1 Department of Civil Engineering, Technichal University of Denmark, 2800 Kgs. Lyngby, Denmark; 2 Faculty of Civil and Environmental Engineering, Technion-Israel Institute of Technology, Haifa 32000, Israel

**Keywords:** distributed diber-optic sensing, direct simple shear, geotechnical engineering, contact problems, soil properties

## Abstract

This paper presents an approach for evaluating the horizontal stresses that develop in geotechnical Direct Simple Shear (DSS) tests through the use of high-resolution distributed fiber optic sensing. For this aim, fiber optics were embedded in 3D printed rings used for confining the soil in the test procedure. An analytical approach linking the measured spatially-distributed strain profile and the internal soil-ring contact stresses is developed in the paper. The method is based on representation of the contact stresses by a Fourier series expansion, and determining the coefficients of the series by minimizing the difference between the measured strain and the analytical strain within the linear elastic ring. The minimization problem results in a linear set of equations that can easily be solved for a given measurement. The approach is demonstrated on a set of drained DSS tests on clean sand specimens. Stress paths using the evaluated horizontal stresses are plotted together with Mohr circles at failure. These illustrate how, in these specific tests, the horizontal stress increases and principal stress direction rotates, until failure occurs along horizontal planes.

## 1. Introduction

Distributed fiber optic sensing has been recognized as one of the potential technologies that could revolutionize structural and geotechnical monitoring and testing (e.g., [1,2,3]). Most of the research in the area has focused on large-scale problems, such as pile foundations [4,5,6,7], pipeline stressing [8,9,10,11], stressing and deformation of secant pile walls [12,13], landslides and sinkholes [14,15,16,17], tunneling stressing [18,19,20], strain measurement and detection of cracks in asphalt pavements [21,22,23,24], and evaluation of tunneling-induced ground deformations [25,26,27]. These applications are based on static, and low (~1 m) sensing resolution limiting themselves to large-scale problems.

New sensing developments, however, allow for higher spatial resolution of a few millimeters to centimeters [28,29,30,31,32]. These developments open the window to new possibilities of smaller-scale applications, such as on-sample measurements [33], high-resolution profiling [34], and key-point characterization methods [35].

**Figure 1:**
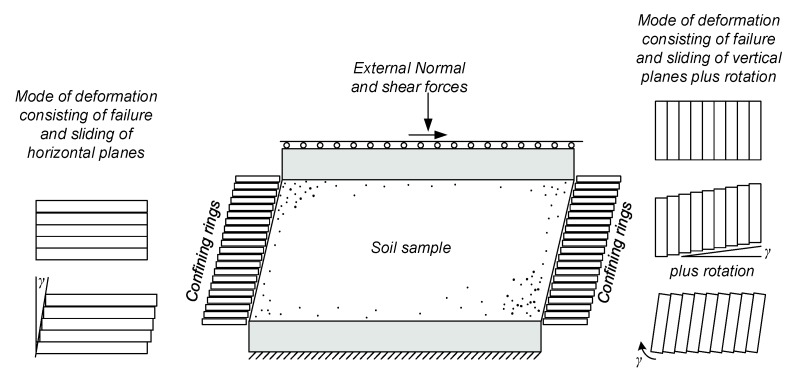
Illustration of the direct simple shear test and possible kinematically compatible modes of deformation (following [44,45]).

This paper suggests an additional new application for high-resolution fiber optic sensing, aiming to enrich the information obtained from a Direct Simple Shear (DSS) test. The DSS test is a common soil laboratory test used to evaluate the geotechnical properties of geomaterials. In the test, a soil sample is sheared in a simple shear mode, such that parallel planes remain parallel and maintain a constant distance, while translating relative to each other (as demonstrated in Figure 1). Guidelines for performing the tests can be found in ASTM D6528-18 [36]. While it is traditionally assumed that failure and the associated deformation occurs due to sliding of horizontal planes, other modes of shearing can lead to kinematically compatible deformations, as illustrated in Figure 1.

A DSS test can be performed either under fixed vertical stress (allowing the soil to compact or dilate), or under a fixed vertical strain (in which case the vertical stress is measured and may decrease or increase). Overall, four parameters are controlled or monitored in the test procedure, namely: the shear strain γ, the shear stress τ, the vertical stress $\sigma_{v}$, and the vertical strain $\epsilon_{v}$ (which supposedly constitutes the volumetric strain in this specific mode of shearing). Two types of DSS devices exist—the first being the complex, non-commercial, Cambridge cuboidal simple shear apparatus [37], and the other the Geonor DSS device [38], which uses a cylindrical specimen confined by a wire-reinforced membrane. As the complete stress condition is not known due to the rotation of principal stresses and strains (not even sufficient to construct a Mohr circle of stresses), measurements need to be performed to evaluate the value of the horizontal stresses at the time of the shearing.

This is achieved by load cells [39] in the Cambridge device, while a resistance wire has been sometimes applied [40] to the Geonor device to evaluate the average radial stress using a Wheatstone bridge. However, typical DSS devices that are available commercially do not have these capabilities. This hinders the interpretation of the test results, as certain assumptions need to be made regarding the stress state at the time of failure.

Over the years, various models have been suggested to analyze the test results based on a mix of analytical and empirical relations. For example, semi-empirical expressions for calculating the horizontal stress, based on the direct proportionality between the inclination of the principal stress axes and the shear stress ratio, were developed by [39,41]. The assumption of collinearity between stresses and strains was used by [42] to develop an analytical incremental expression that allows evaluation of the horizontal stress in DSS devices. Coupled 3D computer simulations of DSS with the measured test response were used by [43] to calibrate the constitutive behavior.

In general, shear tests are used to establish soil stiffness, strength, and shear-volumetric relations (e.g., dilation). These may be a function of the stress level, stress ratios, and strains, which generally constitute the variables of a constitutive model for soil representation. Out of six stress components, only four components are known in typical DSS devices (two measured and two being zero, due to the symmetry of the device and loading). Evaluation of the horizontal stresses can enrich the knowledge to six stress components, and thus allow better interpretation of the results. This may include the evaluation of the confining stress and its effect on the response, the specific effect of the intermediate stress, as well as stress path analysis and its relation to the mechanism of failure, all of which are currently missing from standard DSS devices. Therefore, it is clear that measurements of the horizontal stresses within the sample could contribute to the interpretation of the test results, regardless of the exact test conditions (i.e., drained, undrained, monotonic, or cyclic) or type of soil (i.e., sands or clays).

This paper suggests a new approach for evaluating the horizontal stresses in the sample, and presents the first steps of its development. The approach utilizes recent advances in distributed fiber optic sensing technologies that allow for high accuracy and resolution sensing, together with an approach for determination of the contact stresses. More specifically, the approach links between spatially continuous profiles of the developed strain in the apparatus confining rings with an analytical solution of the rings’ response to contact stresses. The contact stress is resolved by an optimization problem for which the solution can be obtained by a set of linear equations. The paper presents both the analytical development, as well as a demonstration of the approach on the DSS apparatus.

The paper is composed of five main sections. Firstly, the approach of evaluating the contact stresses by an optimization problem of the measured high resolution distributed strain profile linked to the analytical solution of the elastic response of the rings is presented. Secondly, the upgraded apparatus, including fiber-embedded 3D printed rings, is described. Thirdly, test results of the direct simple shear on clean sand are presented. Fourthly, analysis of the measured strain profile and interpretation of the results are presented. Finally, discussion and conclusions are provided.

## 2. Fourier-Based Optimization for Evaluating Contact Stresses

It is assumed that the contact between the membrane (enclosing the soil) and the confining rings in the DSS apparatus is smooth, and that the membrane is sufficiently flexible not to confine the soil by itself, allowing for the contact stress on the ring to be equal to the stresses in the soil. Under this condition, the horizontal traction between the soil and the confining rings is made of normal stresses.

**Figure 2:**
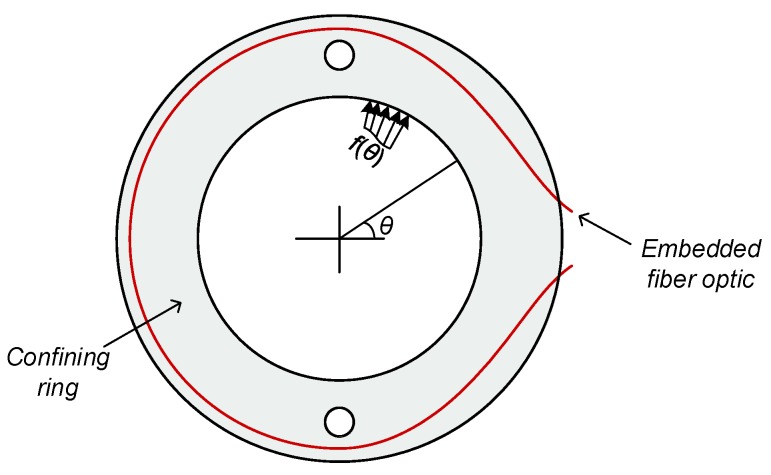
Illustration of a confining ring involved in a direct simple shear device.

Let us focus on the behavior of a given ring within the direct simple shear apparatus, and represent the normal stress acting on the ring by a Fourier series:

(1) $$f\left(\theta \right)=a_{0}+\sum_{i=2}^{N}a_{i}\cos\left(i\theta \right)$$

where f(θ) is the normal stress acting at an angle θ, as illustrated in Figure 2 which represents the plain view of a single ring. $a_{i}$ are the amplitudes of the sinusoids composing the Fourier series and *N* is the number of terms in the Fourier series. Note that the mode of i=1 is dropped from the series on purpose. This is because it is the only mode that does not satisfy a horizontal equilibrium. All other modes satisfy a horizontal equilibrium, and because there is no external support to the rings (assuming the friction between the rings is negligible), then the first mode must be, by definition, zero (i.e., $\int_{0}^{2\pi }a_{1}\cos\left(\theta \right)\cos\left(\theta \right)=0$ and therefore, $a_{1}=0$). In other words, the above Fourier series satisfies global horizontal equilibrium, irrespective of the coefficients’ value. It is also assumed that a symmetry exists relative to θ=0, considering that the horizontal translation and shearing occur in this direction (otherwise, the Fourier series expansion should be enriched by sine functions).

Considering a linear elastic response of the ring, a function can be established to represent the longitudinal strain response along the fiber optic for a given unit mode (i.e., for a given frequency when the amplitude is unit). Such a representation is feasible due to the superposition principle applicable to linear elastic materials. Let us refer to this function as $\Gamma_{i}\left(x\right)$, such that the strain along the monitored fiber, stretched from x=0 to $x=L_{f}$, is:

(2) $$\epsilon \left(x\right)=a_{0}\Gamma_{0}\left(x\right)+\sum_{i=2}^{N}a_{i}\Gamma_{i}\left(x\right)$$

The Γ functions can be derived analytically if a closed form solution exists, or numerically for a general structure. In the current work, $\Gamma_{i}\left(x\right)$ was established by repeated linear elastic finite element simulations, each of which for a different unit mode of the internal normal stress distribution.

For each case, the $\Gamma_{i}\left(x\right)$ was established by extracting the longitudinal strain value along the fiber arc. This can be preformed either by investigating the local stretching of the arc using the displacement field, or by strain transformation (i.e., $n^{T}En$, where E is the strain tensor presented in a matrix form, and n is a unit vector along the arc). The linear elastic finite element simulations were preformed using the mechanical model in COMSOL Multyphysics [46], taking advantage of its parametric sweep option to automate the process of creating $\Gamma_{i}\left(x\right)$ for the various spatial frequencies of the Fourier series. The ability of COMSOL to incorporate functional input to define loads for the boundary conditions facilitated the process of creating $\Gamma_{i}\left(x\right)$. The simulations were performed under small displacements and small strain definitions, ignoring any geometrical nonlinearity aspect.

**Figure 3:**
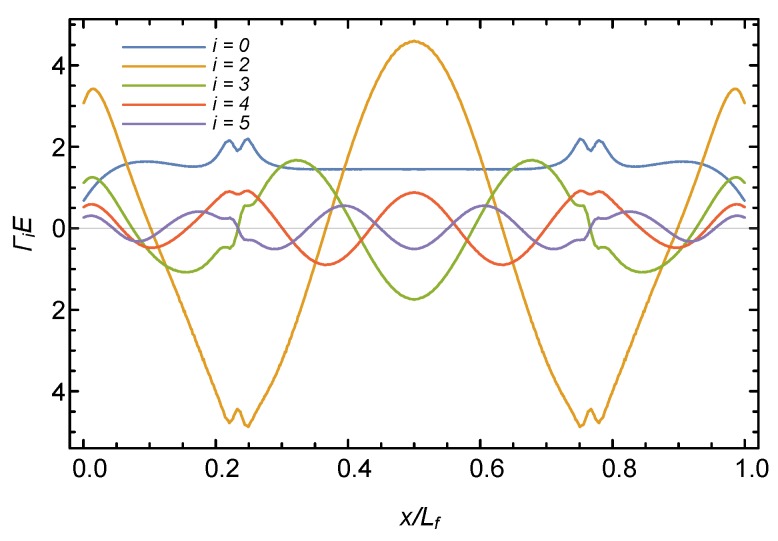
First five modes of Γ functions presented in a normalized manner.

Figure 3 shows the first five modes of $\Gamma_{i}\left(x\right)$ for the location of the fiber plotted in Figure 2, as established by finite element simulations of the 3D printed rings used in the experiments. The embedded fiber length in the ring, $L_{f}$, is equal to 281 mm. The $\Gamma_{i}\left(x\right)$ values in the figure are normalized by Young’s modulus of the ring *E*, assuming a Poisson’s ratio of 0.36, associated with the PLA material from which the rings were fabricated using a 3D printer. The Young’s modulus of the PLA is E=3150 MPa. It can be seen from the figure that the strain response is highly affected by the mode (or spatial frequency), where $\Gamma_{0}$ roughly results in a constant value, and $\Gamma_{5}$ oscillates five times. The effect of the small holes in the ring is also apparent in all modes, at about $x=0.23L_{f}$ and $x=0.77L_{f}$.

In order to evaluate the profile of the contact stresses in an experiment, one may ask what are the best Fourier series coefficients that will lead to an agreement between the measured strain and the analytical strain. This can be formulated mathematically as:

(3) $$a_{i}^{*}=\underset{a_{i}}{\mathrm{argmin}}\int_{0}^{L_{f}}{\left(\epsilon \left(x\right)-\epsilon_{m}\left(x\right)\right)}^{2}dx$$

where ϵ(x) is the analytical strain profile and $\epsilon_{m}\left(x\right)$ is the measured strain profile. Replacing ϵ(x) with the Fourier series (i.e., Equation (2)) and replacing the summation with a dot product of relevant vectors results in:

(4) $$a_{i}^{*}=\underset{a_{i}}{\mathrm{argmin}}\int_{0}^{L_{f}}{\left(a^{T}b-\epsilon_{m}\left(x\right)\right)}^{2}dx=\int_{0}^{L_{f}}a^{T}bb^{T}a-2a^{T}b\epsilon_{m}\left(x\right)+\epsilon_{m}^{2}\left(x\right)dx$$

where $a={\left\{a_{0},a_{2},\ldots ,a_{N}\right\}}^{T}$ and $b={\left\{\Gamma_{0}\left(x\right),\Gamma_{2}\left(x\right),\ldots ,\Gamma_{N}\left(x\right)\right\}}^{T}$. The expression can be further developed to be written in a matrix form:

(5) $$a_{i}^{*}=\underset{a_{i}}{\mathrm{argmin}}\left(a^{T}Ba-2a^{T}b_{1}+C\right)$$

where

(6) $$B=\left[\begin{array}{ccccc} \int_{0}^{L_{f}}\Gamma_{0}\left(x\right)\Gamma_{0}\left(x\right)dx & \int_{0}^{L_{f}}\Gamma_{0}\left(x\right)\Gamma_{2}\left(x\right)dx & \int_{0}^{L_{f}}\Gamma_{0}\left(x\right)\Gamma_{3}\left(x\right)dx & \cdots & \int_{0}^{L_{f}}\Gamma_{0}\left(x\right)\Gamma_{N}\left(x\right)dx\\ \int_{0}^{L_{f}}\Gamma_{2}\left(x\right)\Gamma_{0}\left(x\right)dx & \int_{0}^{L_{f}}\Gamma_{2}\left(x\right)\Gamma_{2}\left(x\right)dx & \int_{0}^{L_{f}}\Gamma_{2}\left(x\right)\Gamma_{3}\left(x\right)dx & \cdots & \int_{0}^{L_{f}}\Gamma_{2}\left(x\right)\Gamma_{N}\left(x\right)dx\\ \vdots & \vdots & \vdots & \ddots & \vdots \\ \int_{0}^{L_{f}}\Gamma_{N}\left(x\right)\Gamma_{0}\left(x\right)dx & \int_{0}^{L_{f}}\Gamma_{N}\left(x\right)\Gamma_{2}\left(x\right)dx & \int_{0}^{L_{f}}\Gamma_{N}\left(x\right)\Gamma_{3}\left(x\right)dx & \cdots & \int_{0}^{L_{f}}\Gamma_{N}\left(x\right)\Gamma_{N}\left(x\right)dx \end{array}\right]$$

and $b_{1}={\left\{\int_{0}^{L_{f}}\Gamma_{0}\left(x\right)\epsilon_{m}\left(x\right)dx,\int_{0}^{L_{f}}\Gamma_{2}\left(x\right)\epsilon_{m}\left(x\right)dx,\ldots ,\int_{0}^{L_{f}}\Gamma_{N}\left(x\right)\epsilon_{m}\left(x\right)dx\right\}}^{T}$. *C* is a constant independent of $a_{i}$ (and equal to $\int_{0}^{L_{f}}\epsilon_{m}^{2}\left(x\right)dx$). Equation (5) is a classical quadratic objective function for which the optimal $a_{i}$ (gathered in vector a) are established through a linear solution, as follows:

(7) $$a=B^{-1}b_{1}$$

Note that matrix B characterizes the fiber-embedded ring (and does not change from one experiment to another), while vector $b_{1}$ includes information from the test itself, embodied in a $\epsilon_{m}\left(x\right)$ profile which is integrated with the various $\Gamma_{i}\left(x\right)$ functions. The above formulation was used in the demonstration experiments, as detailed in the following sections.

## 3. Experimental Setup

**Figure 4:**
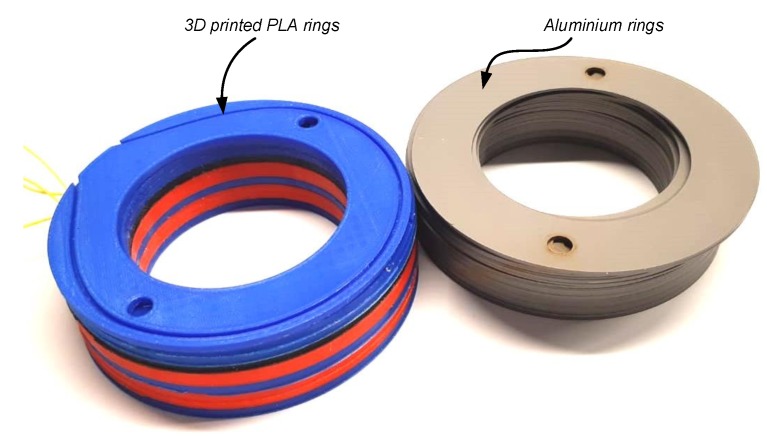
Printed PLA rings next to original Aluminium rings.

**Figure 5:**
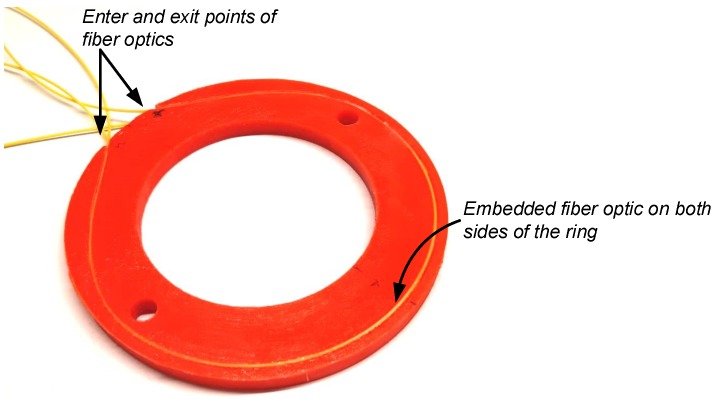
Fiber optic-embedded confining ring.

To demonstrate the approach, the DSS apparatus by the Geocomp Corporation [47] was modified to include fiber-embedded rings. This DSS device uses confining rings to support the soil while sheared, as opposed to an NGI DSS device [38] that uses a reinforced membrane to confine the soil. The inner diameter of the rings is 64.5 mm, and the external diameter is 101.5 mm. Figure 4 shows the original Teflon-coated aluminium rings next to the 3D PLA printed rings. The rings were printed with a thickness of 3 mm, except for the central one which had double the thickness, to provide higher bending resistance. Figure 5 shows a fiber optic embedded ring. A 0.9 mm single mode Corning G652D LSZH tight-buffer fiber optic cable was placed in the 3D printed groove, pre-strained, and glued with a cyanoacrylate adhesive.

The fibers were installed on the top and bottom of the ring. Earlier experiments involved a one-sided, fiber-embedded ring, but it was found that the measurements were sensitive to potential bending of the rings. Consequently, the fibers were installed on both sides of the rings, allowing for compensation of the bending response by summing the strain from both profiles. Other installation configurations are possible, but their investigation is beyond the scope of this paper, which merely presents and demonstrates the new concept.

Note that the solid model used for the 3D printing was also used for generating the $\Gamma_{i}\left(x\right)$ response functions with the finite element code COMSOL Multyphysics [46], as described in the previous section. For the interpretation of the results, the PLA properties were taken as E=3150 MPa following the mechanical properties provided by the technical data sheet of the manufacturer (Ultimaker). The rings were printed under 100% filling configuration to create a true continuum structure.

**Figure 6:**
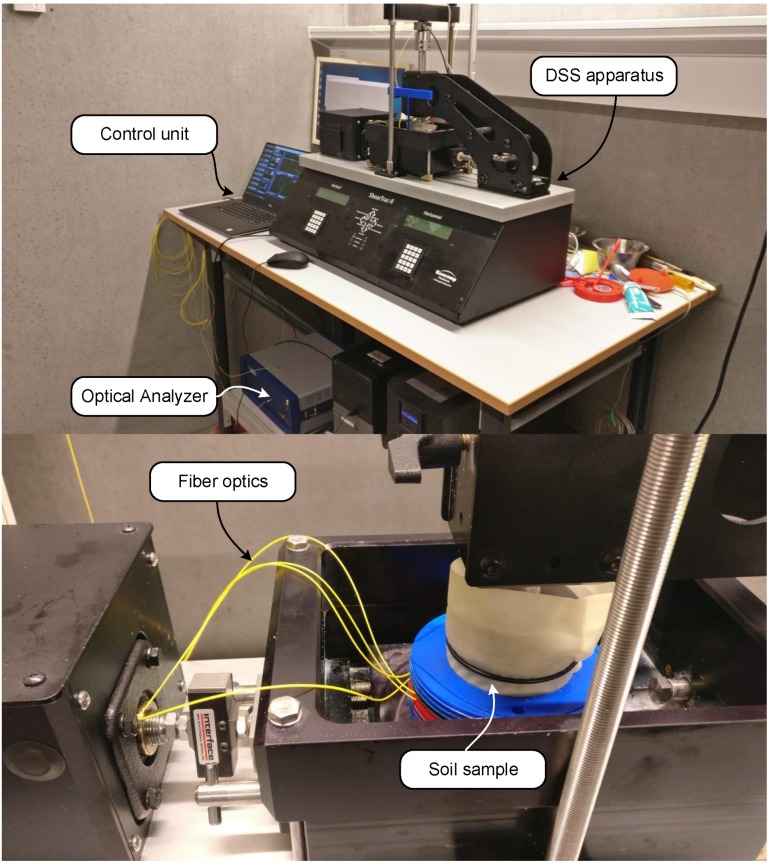
Integrated test system.

Figure 6 shows the integrated test setup. A single fiber-embedded ring was positioned in the middle of the shear sample. The high-resolution strain profiles were recorded with a commercial Optical Backscatter Reflectometer (OBR) device—LUNA 4600 [48]—utilizing the principle of Optical Frequency Domain Reflectometry (OFDR). In this technique, an interferometer is formed by a reference arm internal to the analyzer and the measured fiber optic. By sweeping through the optical frequency range, using a tunable laser, interference fringe data is collected and analyzed both in time and frequency domain to establish changes in length/strain in the fiber. In the current work, strain values were recorded at intervals of 1 mm with a gauge length of 10 mm (i.e., averaged strain over a centered spatial window of 10 mm). In addition to the fiber optic measurements, conventional readings, of horizontal translation, vertical deformation, vertical load, and horizontal forces, were recorded throughout the tests.

## 4. Direct Simple Shear Tests and Results

DSS tests were performed on dry, clean Fontainebleau sand, characterized by quartz particles in the range from 0.063 mm to 0.25 mm, and a uniformity index of U<2 (minimum void ratio of e=0.549 and maximum of e=0.853). Six tests (named T1 to T6 in the paper) were performed on loose- to medium-dense specimens with a relative density ($D_{r})$ roughly ranging over 30% to 50%. The shear tests were performed on normally consolidated samples, loaded to a vertical stress of 485 kPa. Additional tests were performed with the original rings. No significant difference was observed in the overall recorded response of the sample, suggesting that the modifications made to the system did not significantly alter the soil response. Therefore, focus was placed only on the modified system.

**Figure 7:**
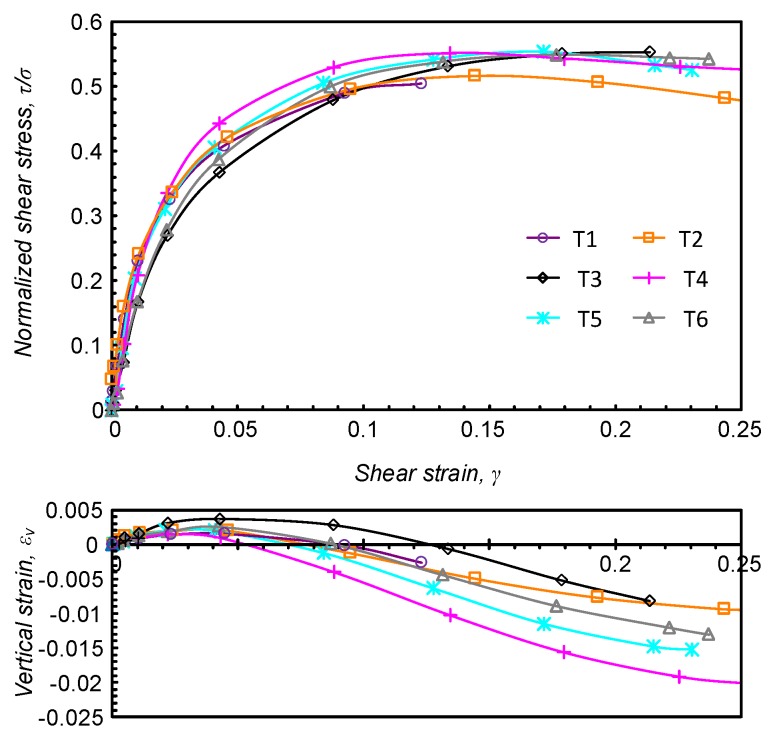
Conventional output from DSS tests.

Figure 7 shows conventional test results in terms of stress-strain curves and dilation response. As can be seen, the stress-strain response is rather similar among the tests, while the volumetric response varies, although all sample showed the same trends of initial contraction followed by dilation.

**Figure 8:**
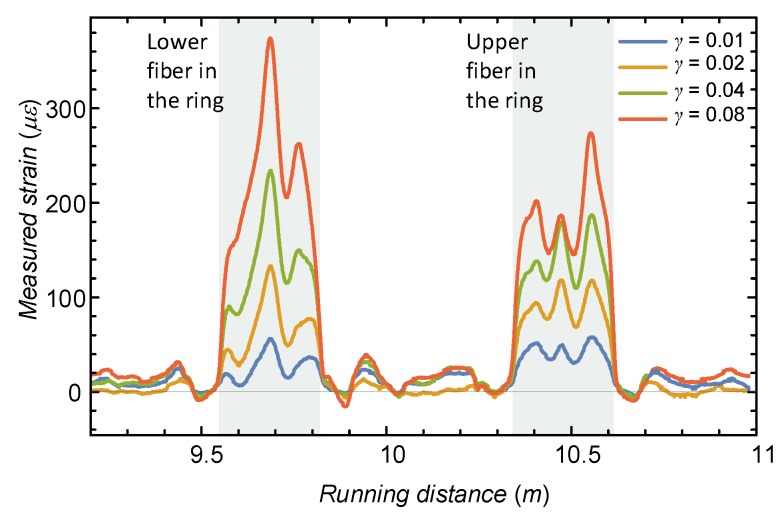
Example of raw measurements along the optical fiber.

Figure 8 shows a typical reading of the developed fiber optic strain in the shearing process (specifically demonstrated for experiment T1). The fiber strain response is also affected by the bending behavior of the ring, and therefore, a post-processing stage was performed in which the fibers were superimposed to nullify (or at least reduce) the bending contribution before they were introduced to the stress evaluation algorithm (which does not include such modes of deformations). Strain changes also appeared outside the ring, although to a much smaller extent. These relate to the fact that the base of the carriage translates and forces movement upon the “free” fiber inside the box, as well as outside (leading to minute displacements and small strains outside the ring area). Note that no temperature compensation was considered, as the experiments were performed in a temperature controlled room.

The strain data was accumulated for the six tests, with measurements being taken at translation intervals of about 0.1 mm until overall translation of 3 mm, and 0.5 mm intervals until translation of 8 mm. The analysis and interpretation of the results are provided in the following section.

## 5. Interpretation of the Test Results and Discussion

The interpretation here focuses on the stress condition at the time of shearing, assuming that a $K_{0}$ condition prevailed prior to shearing. A $K_{0}$ value of 0.54 was considered using Jaky’s formula ($K_{0}=1-\sin\phi^{\prime }$) [49], based on the measured friction angle of the sand, leading to an initial horizontal stress of 264 kPa.

**Figure 9:**
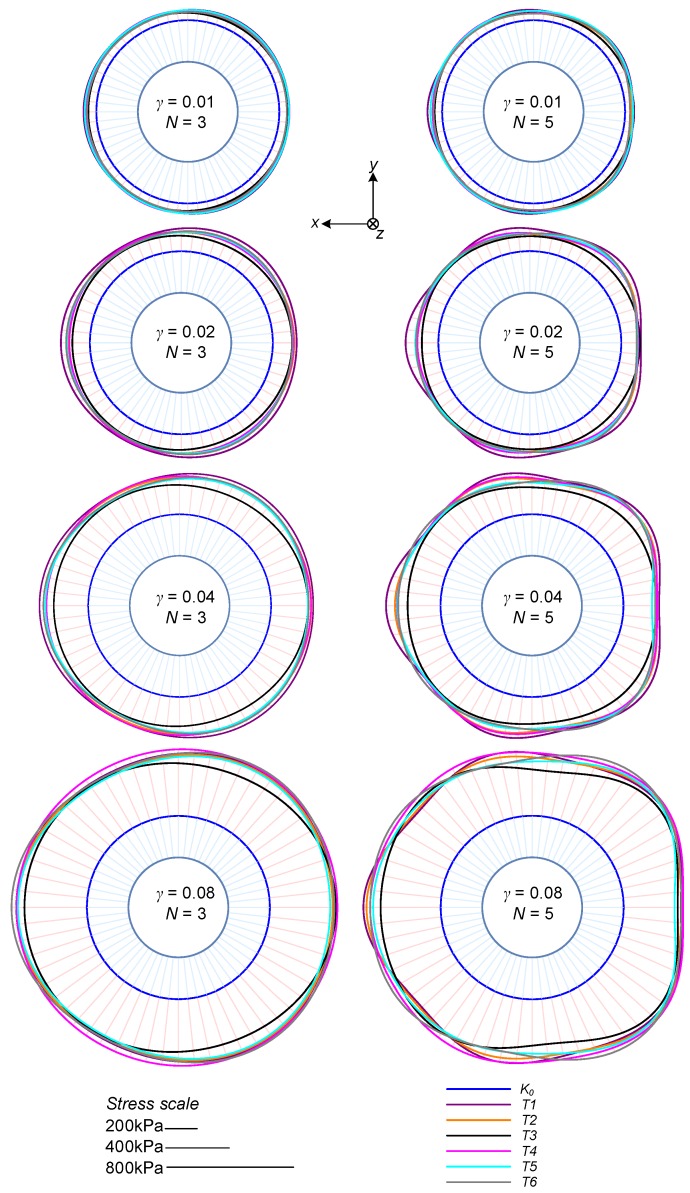
Contact stress distributions at different stages of the shear tests.

Figure 9 shows a graphical representation of the horizontal stress distribution applied to the soil sample, as analyzed by the Fourier-based optimization at different stages of testing, expressed by the shear strain, γ.

Specifically, values at γ= 0.01, 0.02, 0.04, and 0.08 were plotted based on analyses with 3 and 5 modes. The radial distance from the center circle represents the amplitude of the horizontal normal stress to the soil sample (equal to the stress applied to the confining ring). The $K_{0}$ (initial) condition is marked by a blue line, where the length of the light blue arrows represents its magnitude. A scale is given for reference, and the light red arrows extend to the largest calculated stress. Let us refer to a Cartesian coordinate system in which *z* represents the vertical direction, and *x* the direction of translation (*y* being the other horizontal direction). These are plotted in the figure.

As can be seen, with increasing shear strain, the horizontal stress increases in all directions. Overall, the magnitude of the developed stress profiles and their distribution are similar among the different tests. The magnitude of variation is similar to that observed in the applied shear stress in Figure 7. The analysis with five modes shows greater discrepancy between the tests, specifically in the higher spatial frequencies. This is because higher frequencies are more sensitive to measurement errors. While the five modes analysis indicates a “square” type of distribution, the stress values in the translation direction ($\sigma_{x}$) and the perpendicular one ($\sigma_{y}$) are rather similar to those based on the three modes analysis. Hereafter, all analyses are performed with the five modes data, although similar results and trends are observed from the three modes analyses.

**Figure 10:**
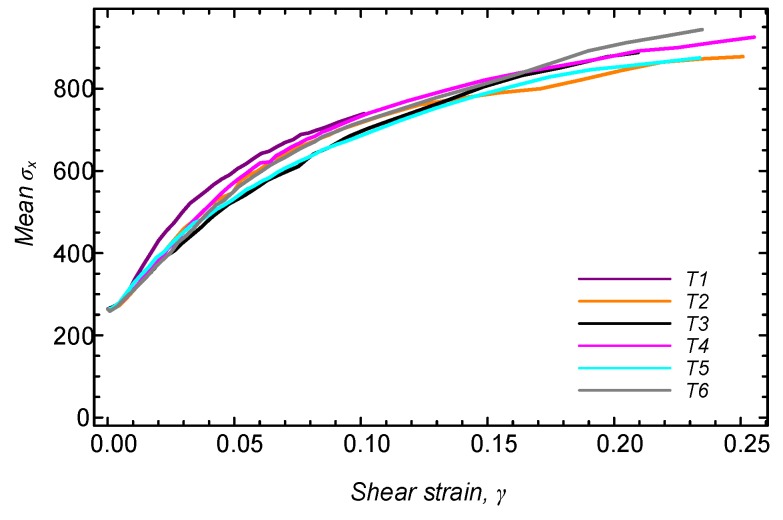
Mean $\sigma_{x}$ throughout the tests, taken as 0.5(f(θ=0)+f(θ=π)).

Figure 10 shows the development of the mean $\sigma_{x}$, taken as 0.5(f(θ=0)+f(θ=π)). As can be seen, as the shearing process progresses, the horizontal stress increases, eventually becoming larger than the vertical stress (equal to 485 kPa) by a factor of almost two.

Assuming that this mean $\sigma_{x}$ represents the average normal stress applied to sheared vertical planes, a stress path can be plotted in terms of $\left(\sigma_{x},\tau_{xz}\right)$. This stress path is unknown from conventional DSS testing, and is a result of the new suggested approach. It can supplement the conventional $\left(\sigma_{z},\tau_{zx}\right)$ stress path to allow constructions of Mohr stress circles throughout the shearing process.

**Figure 11:**
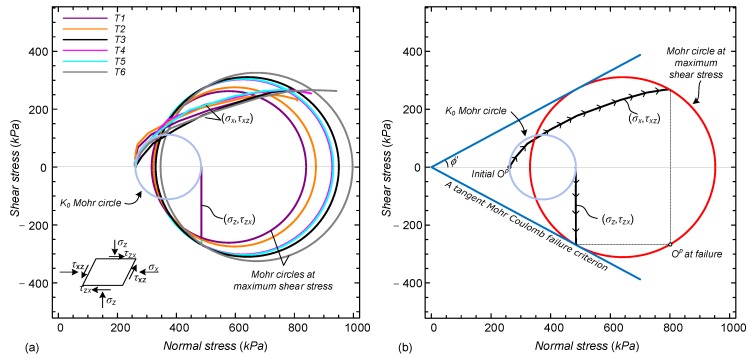
(**a**) ($\sigma_{z},\tau_{zx}$) and ($\sigma_{x},\tau_{xz}$) stress paths for all tests; (**b**) interpretation illustrated on T3.

Figure 11a shows the various stress paths established from the analysis of all tests. The Mohr circles associated with the peak shear stress, $\tau_{zx}$, are presented, as well as that of the initial $K_{0}$ condition (identical for all samples). The $\left(\sigma_{z},\tau_{zx}\right)$ stress path is essentially identical for all samples, with a slight variation in the peak $\tau_{zx}$ value (as also demonstrated in stress-strain curves in Figure 7). The interpretation of the different samples is rather similar, and as an example, Figure 11b shows the interpretation for test T3, entailing the stress paths, initial and final Mohr circles, their poles, and a tangent Mohr-Coulomb cohesionless failure criterion.

It can clearly be seen that the $\left(\sigma_{z},\tau_{zx}\right)$ stress path reaches the Mohr-Coulomb failure line at tangent point (although this condition was not forced). This indicates that failure occurred along horizontal planes. The principal stress directions rotated significantly throughout the shearing process of the sample, by about 60 degrees, leading the pole to change its position (marked in the figure as “initial $O^{p}$” and “$O^{p}$ at failure”).

Repeating the same process on the other samples resulted in the same trends. The obtained friction angles, by the tangent line to the Mohr circle of each test, ranged from 27 to 29 degrees among the test. These values are similar to those obtained with the conventional setup and slightly less than those measured on a range of sands in direct shear [50].

The fact that the ($\sigma_{z},\tau_{zx}$) stress path reaches the failure envelope at the same location as the tangent to the Mohr circle infers that a direct evaluation of the strength properties from a conventional analysis assuming $\tau_{zx}/\sigma_{z}=\tan\phi^{\prime }$ is appropriate for these specific tests. However, this may not always be the case, especially in undrained (or zero volumetric strain) conditions, where failure occurs along vertical planes and the greatest shear stress experienced does not correspond to the maximum shear stress measured [44]. The ability to evaluate such a stress path development, as well as rotation in principal directions, may be important for analysis of undrained shearing and cyclic loading, where there might be interest in the change of confining mean stress due to compaction and dilation at shear direction reversal [51].

## 6. Conclusions

This paper presented a new approach for evaluating the horizontal stress condition in direct simple shear tests using high-resolution fiber optic sensing coupled to a Fourier based elastic solution of the confining rings. An optimization problem was formulated aiming to evaluate the best stress profile (in terms of Fourier coefficients) generating an analytical stress profile that would match the measured strain profile.

The approach was demonstrated with a commercial DSS apparatus, for which a new set of fiber optic-embedded confining rings was produced using 3D printing. Six drained direct simple shear tests were performed on clean sand to demonstrate the applicability of the approach. It was demonstrated how a stress path and Mohr circles can be evaluated with the new information derived from fiber optic sensing. Specifically, it was shown that in these particular (drained) tests, the soil truly fails along horizontal plane when considering a Mohr Coulomb yield condition.

The main focus of the paper was to conceptually develop a suitable approach for utilizing high-resolution fiber optic sensing for laboratory geotechnical testing, and to demonstrate its feasibility. Clearly, further research could be performed to improve the suggested approach. For example, studies could be performed to seek the best position of the optical fiber within the rings. Investigation of different ring materials or shape could be considered, as well as utilization of other high-resolution sensing techniques. These investigations, as well as investigations into more complex soil behavior, are beyond the scope of this paper, which simply attempts to take the first steps of advancing the use of high-resolution fiber optic sensing for laboratory geotechnical testing.
